# Disparity in the timing of vertebrate diversification events between the northern and southern hemispheres

**DOI:** 10.1186/1471-2148-12-244

**Published:** 2012-12-15

**Authors:** Reid Tingley, Sylvain Dubey

**Affiliations:** 1 School of Botany, University of Melbourne, Melbourne, VIC, 3010, Australia; 2 Department of Ecology and Evolution, Biophore Bld, University of Lausanne, Lausanne, 1015, Switzerland

**Keywords:** Climate change, Dispersal, Ectotherm, Endotherm, Genetic diversity, Glaciation, Intraspecific diversification, Quaternary

## Abstract

**Background:**

Climatic oscillations throughout the Quaternary had profound effects on temperate biodiversity, but the extent of Quaternary climate change was more severe in temperate regions of the northern hemisphere than in the southern hemisphere. We sought to determine whether this geographic disparity differentially influenced the timing of intraspecific diversification events within ectothermic and endothermic vertebrate species. Using published phylogenetic hypotheses, we gathered data on the oldest intraspecific diversification event within mammal, bird, freshwater fish, amphibian, and reptile species from temperate-zone areas. We then tested whether the timing of diversification events differed between hemispheres.

**Results:**

Our analyses provide strong evidence that vertebrates from temperate regions of the northern hemisphere are younger than those from the southern hemisphere. However, we find little evidence to suggest that this relationship differs between endotherms versus ectotherms, or that it varies widely across the five classes of vertebrates that we considered. In addition, we find that on average, endothermic species are much younger than ectothermic species.

**Conclusion:**

Our findings suggest that geographic variation in the magnitude of climatic oscillations during the Quaternary led to substantial disparity in the timing of intraspecific diversification events between northern and southern hemisphere vertebrates, and that the magnitude of this divergence is largely congruent across vertebrate taxa.

## Background

**Figure 1:**
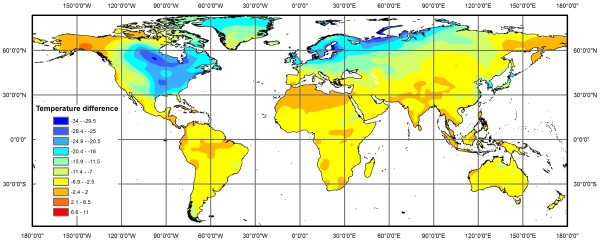
**Difference in mean annual temperature between the last glacial maximum (LGM; ~21 ka years before present) and present day climate (1950-2000).** LGM predictions were based on the CCSM global circulation model ([10]; data available from http://www.worldclim.org). Although many intraspecific diversification events within vertebrates occurred much earlier than the LGM, the change in temperature between the LGM and present is representative of climatic oscillations over much longer timescales [11].

Investigating how historic changes in climatic conditions influenced the geographic distributions of species provides unique opportunities to understand how extinction and dispersal influence patterns of contemporary biodiversity, and can provide novel insights into how species will shift their distributions in response to contemporary climate change. Climate cooling began in the lower Miocene (~10 million years ago) [1,2], and the Pliocene transition marked the disappearance of stable climatic conditions throughout the globe (see e.g. [1] for a review). However, the onset of significant climatic oscillations began in the Quaternary (~1.8 million years ago to present), and these changes had particularly profound effects on temperate biodiversity [3-5]. In many temperate regions, the magnitude of these fluctuations is manifested in the genetic structure of extant populations [6], but the severity of Quaternary climate change was not the same in temperate regions of the northern and southern hemispheres (see Figure 1). Extensive ice sheets were almost entirely absent in the southern hemisphere, whereas large portions of North America and Eurasia were covered by glaciers and permafrost for extended periods throughout the Quaternary [1,7]. Consequently, the contemporary distributions of many northern hemisphere vertebrates reflect relatively recent recolonisations of previously unsuitable areas from climatic refugia [6,8]. This pattern of extinction and recolonisation should have, in turn, constrained intraspecific divergences between genetic lineages to more recent timescales in the northern hemisphere. Indeed, the contraction of species to refugia led to a loss of genetic diversity and intraspecific genetic lineages [9], which could have eliminated ancient lineages and resulted in more recent diversification events in the northern hemisphere. 

Recent meta-analyses based on molecular dating [12,13] have revealed significant differences in the timing of the oldest intraspecific diversification event within amphibian and reptile species from the northern and southern hemispheres. In both groups, northern hemisphere species were found to be younger than southern hemisphere species. Similarly, splits between sister species occurred more recently in the northern hemisphere than in southern hemisphere [13].

A fundamental question is whether this pattern is widespread in vertebrates, or whether it is a pattern that is specific to ectothermic taxa. Ectothermic vertebrates depend on abiotic conditions to thermoregulate, and thus are extremely vulnerable to climatic fluctuations [14,15]. For example, temperature-dependent sex determination is widespread in reptiles such as lizards, turtles, and crocodiles, making these taxa particularly sensitive to abrupt variations in ambient temperature [16]. This thermal sensitivity means that populations of ectotherms may be more likely to survive (and thus exhibit greater genetic differentiation) in regions with stable climatic conditions. In addition, ectothermic vertebrates such as amphibians, reptiles, and freshwater fish generally have poor dispersal abilities in comparison to similarly-sized endotherms such as mammals and birds, which could expose ectotherms to more severe climates at a given location. Overall, we therefore might expect the ages of ectothermic vertebrates to show a greater degree of disparity between hemispheres than we would for endotherms [17].

In the present study, we sought to determine whether geographic disparity in Quaternary climatic oscillations between the northern and southern hemispheres differentially influenced the timing of intraspecific diversification events within vertebrate species. We then tested whether the relationship between hemisphere of origin and the timing of intraspecific diversification differed between ectotherms versus endotherms.

## Results

**Figure 2:**
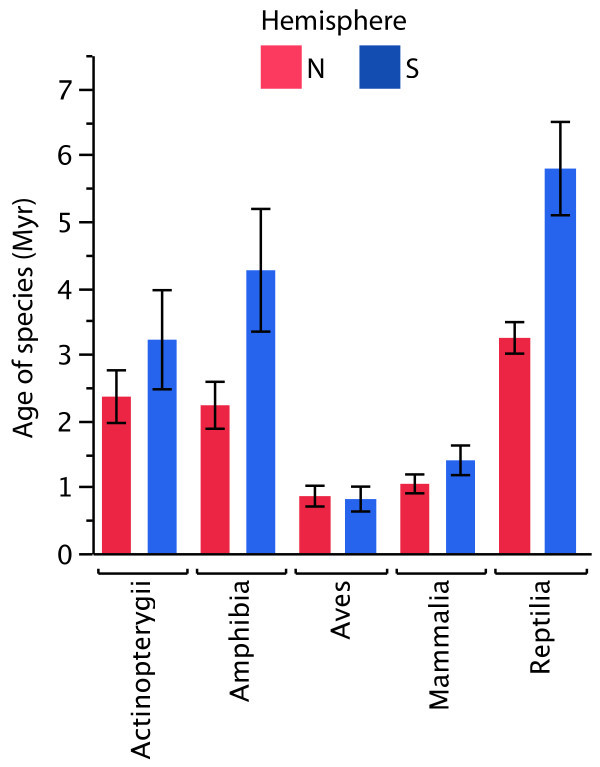
**Disparity in the oldest estimated dates of intraspecific diversification events within vertebrate species from temperate regions of the northern and southern hemispheres.** The figure shows means and standard errors of species ages (millions of years ago) of northern hemisphere versus southern hemisphere vertebrates in each class (Actinopterygii, Amphibia, Aves, Mammalia, and Reptilia).

Our analyses provide strong evidence that vertebrates from temperate regions of the northern hemisphere are younger than those from the southern hemisphere (Figure 2; Table 1a). Importantly, this disparity between hemispheres was not due to a latitudinal bias in the origins of the species included in our analyses. Restricting our analyses to high- (Table 1b) and low-latitude (Table 1c) species did not influence our finding that diversification events occurred more recently in the northern hemisphere. Overall, there was no evidence to suggest that there was an interaction between taxonomic class and hemisphere (Table 1), indicating that the relationship between species age and geographic location was relatively consistent across all five classes of vertebrates that we considered. Disparity in the timing of diversification events between hemispheres was greatest for reptiles, but excluding reptiles from our analyses did not qualitatively change any of our conclusions.

**Table 1:** Effects of hemisphere of origin (northern versus southern) and taxonomic class (Mammalia, Aves, Amphibia, Reptilia, Actinopterygii) on intraspecific estimates of species ages from temperate-regions across the globe

**Variable**	**Likelihood ratio (*D*)**	**P**
(a) *Complete dataset*		
Hemisphere	*D* = 14.2	< 0.001
Taxonomic class	*D* = 28.8	< 0.0001
Hemisphere * Taxonomic class	*D* = 1.72	0.786
(b) *High-latitude species*		
Hemisphere	*D* = 10.5	0.00120
Taxonomic class	*D* = 15.0	0.00190
Hemisphere * Taxonomic class	*D* = 2.14	0.544
(c) *Low-latitude species*		
Hemisphere	*D* = 9.62	0.00190
Taxonomic class	*D* = 63.5	< 0.0001
Hemisphere * Taxonomic class	*D* = 7.17	0.127

Results are shown for the entire dataset (a), for the subset of species that are distributed above 40° N or S (b), and for the subset of species that are distributed between the tropics and 40° N or S (c). Likelihood ratio tests were conducted between nested models that included and excluded each variable; however, main effects were tested for significance only after excluding the interaction term.

When each class was analyzed separately, only the mean ages of reptiles (likelihood ratio test between models including and excluding hemisphere effect: *D* = 8.22, *P* = 0.00410) and mammals (*D* = 4.040, *P* = 0.0443) differed between hemispheres. There were no significant effects of hemisphere of origin on the mean ages of birds (*D* = 1.33, *P* = 0.248), fishes (*D* = 0.903, *P* = 0.342), or amphibians (*D* = 1.79, *P* = 0.181). Nevertheless, on average, fishes and amphibians from the southern hemisphere were older than those from the northern hemisphere (Figure 2). Furthermore, our finding that diversification events occurred more recently in the southern hemisphere in birds (Figure 2) was highly dependent on the inclusion of a single northern hemisphere species (*Alcippe morrisonia*) with highly divergent populations (9.86 million years). When this species was excluded from our analyses, bird species from the southern hemisphere were older (mean ± SE 0.814 ± 0.187) than those from the northern hemisphere (0.736 ± 0.0999), although differences between hemispheres remained non-significant (*D* = 1.78, *P* = 0.182). Interestingly, our analysis also revealed strong differences between the mean ages of the five vertebrate classes (Figure 2; Table 1).

Similar results were obtained when we compared species differing in their thermoregulatory strategies (i.e., endotherms versus ectotherms). Ectotherms were older than endotherms, and vertebrates that originated from the northern hemisphere were younger than those from the southern hemisphere, but the relationship between species age and hemisphere of origin did not differ between ectotherms and endotherms (Table 2a). Restricting our analyses to high- (Table 2b) and low-latitude (Table 2c) species did not substantially influence these conclusions. When each group was analyzed separately, both endotherms (*D* = 5.76, *P* = 0.0164) and ectotherms (*D* = 10.6, *P* = 0.00110) from the southern hemisphere were significantly older than those from the northern hemisphere. 

**Table 2:** Effects of hemisphere (northern versus southern) and thermoregulatory strategy (endotherm versus ectotherm) on intraspecific estimates of species ages from temperate-regions across the globe

**Variable**	**Likelihood ratio (*D*)**	**P**
(a) *Complete dataset*		
Hemisphere	*D* = 14.1	< 0.001
Thermoregulatory strategy	*D* = 16.1	< 0.0001
Hemisphere * Thermoregulatory strategy	*D* = 0.844	0.358
(b) *High-latitude species*		
Hemisphere	*D* = 13.2	< 0.001
Thermoregulatory strategy	*D* = 7.35	0.00670
Hemisphere * Thermoregulatory strategy	*D* = 0.521	0.471
(c) *Low-latitude species*		
Hemisphere	*D* = 8.98	0.00270
Thermoregulatory strategy	*D* = 60.7	< 0.0001
Hemisphere * Thermoregulatory strategy	*D* = 0.652	0.419

Results are shown for the entire dataset (a), for the subset of species that are distributed above 40° N or S (b), and for the subset of species that are distributed between the tropics and 40° N or S (c). Likelihood ratio tests were conducted between nested models that included and excluded each variable; however, main effects were tested for significance only after excluding the interaction term.

## Discussion

Our analysis of molecular phylogenies provides compelling evidence that vertebrate diversification events occurred much more recently in the northern hemisphere than in the southern hemisphere. This pattern was remarkably consistent across different latitudinal subsets of species, suggesting that the geographic variation in species’ ages that we observed here was not substantially influenced by the exclusion of species that were distributed solely at low or high latitudes.

Why are vertebrates from the northern hemisphere younger than those from the southern hemisphere? One plausible explanation, as outlined in the Introduction, is that the observed disparity in diversification histories reflects geographic differences in the extent of climatically-driven extinction events that occurred throughout the Quaternary. Some regions of the southern hemisphere (e.g., inland Australia) were also severely affected by dry conditions during the Quaternary [11], but palynological data indicate that forested and partially forested areas still persisted in coastal regions, even during the Last Glacial Maximum [3]. Although lineages of some of the species used in our analyses diverged prior to the Quaternary, this does not mean that climatic oscillations during the Quaternary could not be responsible for the observed patterns. Many species lost genetic lineages during the Quaternary [9], and thus a species may have (randomly) lost its oldest lineage while undergoing range contraction in response to Quaternary climate change. For example, a species that had similar levels of genetic differentiation in both hemispheres prior to the Quaternary would have had a higher probability of losing its oldest genetic lineage in the northern hemisphere because the magnitude of Quaternary climate change was greater there.

Our findings accord with the results of previous analyses that focused exclusively on amphibians and reptiles [12,13], and thus demonstrate that the patterns revealed by these earlier studies apply to vertebrates as a whole. Indeed, we found no evidence for an interaction between taxonomic class and hemisphere of origin, suggesting that the relationship between geographic origin and species age was broadly consistent across the five classes of vertebrates included in our analyses. Although differences between hemispheres were only statistically significant for mammals and reptiles when each taxonomic group was analyzed separately, partitioning our dataset by taxonomic group entailed a considerable loss of statistical power. In addition, the regression coefficients describing the effects of hemisphere of origin were in the same direction for four out of five taxa, the exception being birds. Nevertheless, similar results were obtained for birds when a single northern hemisphere species that had exceptionally divergent populations was removed from our analyses.

At first glance, this consistency in the effects of geographic location on diversification histories is somewhat surprising given the marked differences in dispersal abilities between the taxa included in our analyses [17,18]. One potential reason for this seemingly counterintuitive result is that differences in dispersal abilities between taxa are trivial in comparison to the disparity in Quaternary climate changes between the northern and southern hemispheres. However, it should be noted that the mean age of birds, a group that has the greatest dispersal abilities of the taxonomic classes that we considered, showed the least disparity between hemispheres.

Interestingly, studies of antbirds and passerines have revealed greater genetic distances (based on allozymes) within neotropical species than in species from the Nearctic [19,20]. Similar patterns have also been documented in a suite of birds and mammals from the New World [21], as well as in plants [22] and vertebrates [23] distributed across the globe. Collectively, the results of these studies suggest greater intraspecific genetic variation in regions that have experienced stable long-term climatic conditions. Thus, our finding that diversification events occurred earlier in the southern hemisphere, where climatic oscillations have also been less severe in the past, is in broad accordance with earlier studies on a wide range of taxa.

We also found that the relationship between hemisphere of origin and species age did not differ between ectotherms and endotherms, and this pattern was also evident when we considered each of these two groups in isolation. Future research could usefully explore whether species-level traits that do not show a strong taxonomic signal (e.g., habitat-use) explain additional geographic variation in species ages [13].

However, we cannot dismiss the possibility that our findings may be an artifact of differing levels of taxonomic knowledge about the vertebrate faunas of the northern and southern hemispheres [13,20]. Species from the southern hemisphere have generally been less intensively studied than species from the northern hemisphere, and thus some southern hemisphere ‘species’ included in our analyses may actually represent composite taxa. This taxonomic ignorance would, in turn, result in over-estimations of species’ ages in the southern hemisphere. Dubey & Shine [13] demonstrated that geographic disparity in taxonomic ignorance is a relatively poor explanation for differences in the ages of amphibian species between hemispheres, but that this mechanism could potentially explain the different diversification histories of the two hemispheres for reptiles. However, we consider this interpretation less likely than climatically-driven extinction events given that our findings were relatively consistent across such broadly divergent taxa (see above). We further consider it unlikely that our results are an artifact of using intraspecific rather than interspecific divergence times, given that Dubey & Shine [13] found very similar geographic patterns in species ages regardless of whether the analysis was performed with intraspecific divergences (across orders and suborders) or with the cladogenic events responsible for species formation [13]. Thus, the same parameters appear to influence these two types of measures [13]. Our finding that diversification events occurred earlier in the southern hemisphere may also have been influenced by the restricted distributions of some taxonomic groups throughout the globe. Some taxa are strictly or more widely represented in one hemisphere, which could impact species age estimations (considering that each taxa exhibits different life-history traits and characteristics).

Our analyses also revealed differences in the timing of intraspecific diversification events between taxonomic classes, and between ectotherms and endotherms. In particular, the mean ages of ectotherms (fishes, amphibians, and reptiles) were older than those of endotherms (mammals and birds). This pattern could be the result of several different mechanisms, and these mechanisms are not mutually exclusive:

i. The differences we observed between endothermic and ectothermic vertebrates could have been due to differences in the dispersal abilities of these groups. Endothermic vertebrates are typically capable of dispersing greater distances than ectothermic vertebrates (e.g., [17,18,24]), and taxa that are capable of dispersing greater distances generally have more homogeneous population structures compared to less vagile taxa. Such homogenization would result in fewer divergent lineages within endothermic vertebrates, and ultimately more recent intraspecific diversification events.

ii. Alternatively, the observed differences between endothermic and ectothermic vertebrates could have been due to differences in energetic demands during hibernation. When abiotic conditions become unfavorable, ectothermic vertebrates are capable of lowering their rates of resource consumption to a far greater degree than are endothermic vertebrates [25-27], and thus ectotherms may have been less adversely affected by harsh winter conditions during the Pleistocene. In addition, many ectothermic vertebrates possess efficient mechanisms to deal with sub-zero temperatures (e.g., freeze-avoidance or freeze tolerance strategies: [28-30]). Hence, ectothermic vertebrates may have been more likely to survive climatic fluctuations during the Quaternary compared to endotherms, at least in locations where the minimum temperature required for ectotherm activity was reached during part of the year.

iii. Finally, differences in the timing of intraspecific diversification events between groups may have been caused by variation in life-history traits. For example, reproductive isolation is faster in particular groups such as mammals (compared to birds and amphibians) due to a higher possible rate of regulatory evolution, causing higher probabilities of developmental incompatibilities between mammal species [31]. Consequently, for the same level of genetic divergence, two lineages could be considered sister species in mammals, and intraspecific lineages in birds or amphibians. The vertebrate classes we examined also differ markedly in a wide array of additional traits (e.g., body size), and such traits are known to impact rates of molecular evolution [32-34].

## Conclusions

We have shown that there is substantial geographic variation in the timing of intraspecific diversification events within temperate-zone vertebrates. Vertebrate diversification events occurred much more recently in the northern than in the southern hemisphere, plausibly reflecting geographic disparity in the magnitude of climatic oscillations that occurred throughout the Quaternary. Importantly, the relationship between diversification history and geographic location did not differ between endotherms versus ectotherms, or across different classes of vertebrates, suggesting that highly divergent taxa responded similarly to past climate change. However, our analyses did reveal considerable variation in the age of the oldest intraspecific diversification event between taxonomic classes, and between ectotherms and endotherms. Thus, diversification histories within vertebrates appear to not only be influenced by abiotic factors such as climatic fluctuations, but also by taxon-specific parameters (e.g., particular life-history traits). Future studies should seek to clarify the roles of such traits in determining spatial and temporal variation in intraspecific diversification events.

## Methods

We gathered data on the oldest intraspecific diversification event within temperate-zone mammal (n = 113), bird (n = 99), amphibian (n = 46), fish (n = 53), and reptile (n = 161) species from published phylogenetic articles using searches within Scopus and ISI Web of Knowledge (see online Additional file 1: Appendix S1; as in [12,35]). The oldest intraspecific diversification event is an estimate of the amount of time since extant populations last shared a common ancestor (i.e. the split between the most basal lineage and the remaining lineages of a given species), and is highly correlated with interspecific estimates of species age [13]. Only data from comprehensive phylogeographic and phylogenetic studies were included in our analyses. Because rates of molecular evolution can vary across taxa [36], we did not convert sequence divergence values provided in original articles into diversification dates using a standard molecular clock (e.g., 2%; [3]). Instead, we included only those studies which explicitly provided a diversification date (although estimated dates were sometimes based on substitution rates from closely related taxa). Within amphibians, urodeles (salamanders and newts) were excluded from our analyses, because no urodele species occupy temperate regions in the southern hemisphere. We also excluded non-monophyletic species (based on recent molecular phylogenetic studies) in order to avoid inaccurate species age estimations.

To test for differences in the timing of diversification events between the northern and southern hemispheres, we used linear mixed effects models with species age (Box-Cox transformed to achieve normality) as the dependent variable, and hemisphere of origin and either taxonomic class or thermoregulatory strategy (ectothermy versus endothermy) as fixed effects. Because the timing of diversification events may not be independent across taxa, we also tested for taxonomic autocorrelation by including taxonomic order as a random effect. Insufficient replication at lower taxonomic levels prohibited us from accounting for finer-scale taxonomic autocorrelation. In cases where variances differed between hemispheres or taxonomic groups (heteroscedasticity), a constant variance structure was used to allow for different spread per stratum. The final structure of each model was determined using likelihood ratio tests (α = 0.05). When taxonomic random effects were non-significant, models were refit using generalized least squares. These analyses were then repeated for each taxonomic group and thermoregulatory strategy separately.

We also examined the consistency of our results by analyzing latitudinal subsets of the broader dataset. Specifically, we tested for a latitudinal bias in the differences in species ages between northern and southern hemispheres by repeating the above analyses on (i) low-latitude species only (distributed between the tropics and |40°|), and (ii) high-latitude species only (distributed above |40°|). Amphibians were not represented among high-latitude species in the southern hemisphere, and thus were removed from the latter of these analyses. It is also important to note that for the southern hemisphere, data for species distributed above 40°S are strictly restricted to Oceania (New Zealand and Tasmania) and South America, as the southern tip of Africa is situated at ~35°S. For birds, we only included species that have breeding ranges exclusively above or below |40°| latitude, rather than using an area-weighted centroid. All statistical analyses were conducted in R^©^ 2.13.0 using the nlme library (R Development Core Team, 2011).

## Competing interests

The authors declare that they have no competing interests.

## Authors’ contributions

SD and RT contributed with the conceptual development of the work and the writing of the manuscript. RT carried out the analyses. All authors read and approved the final version of the manuscript.

## Supplementary Material

Additional file 1 **Appendix S1.** Raw data on the age of species, based on oldest intraspecific diversification events as revealed by molecular phylogenies. The Table shows Species, Hemisphere (N: northern, S: southern), Latitude (below 40°, above 40°, or in both areas), Age (millions of years), and corresponding references. Click here for file
